# Associations between social drivers of health and breast cancer stage at diagnosis among U.S. Black women

**DOI:** 10.1038/s41523-025-00804-0

**Published:** 2025-08-06

**Authors:** Mollie E. Barnard, Bo Qin, Marc A. Emerson, Etienne X. Holder, Matthew R. Dunn, Shromona Sarkar, Nuo N. Xu, Yutong Li, Christine B. Ambrosone, Elisa V. Bandera, Julie R. Palmer, Melissa A. Troester, Terry Hyslop, the Breast Cancer Research Foundation’s Health Equity Initiative, Mollie E. Barnard, Bo Qin, Marc A. Emerson, Etienne X. Holder, Christine B. Ambrosone, Elisa V. Bandera, Julie R. Palmer, Melissa A. Troester, Lori J. Pierce, Lisa A. Carey, Melissa B. Davis, Dawn L. Hershman, Lisa A. Newman, Charles M. Perou, Julienne E. Bower, Scarlett Gomez, Terry Hyslop, Celeste Leigh Pearce, Priya Malhotra, Dorraya El-Ashry, Judy E. Garber, Larry Norton

**Affiliations:** 1 Slone Epidemiology Center, Boston University Chobanian & Avedisian School of Medicine, Boston, MA USA; 2 Rutgers Cancer Institute, New Brunswick, NJ USA; 3 University of North Carolina Lineberger Comprehensive Cancer Center, Chapel Hill, NC USA; 4 Division of Biostatistics/Bioinformatics, Thomas Jefferson University, Philadelphia, PA USA; 5 Roswell Park Comprehensive Cancer Center, Buffalo, NY USA; 6 Sidney Kimmel Comprehensive Cancer Center, Philadelphia, PA USA; 7 University of Michigan, Ann Arbor, MI USA; 8 Morehouse School of Medicine, Riverdale, GA USA; 9 Columbia University Medical Center, New York, NY USA; 10 Weill Cornell Medicine, New York, NY USA; 11 University of California, Jonsson Comprehensive Cancer Center, Los Angeles, CA USA; 12 University of California, San Francisco, San Francisco, CA USA; 13 Breast Cancer Research Foundation, New York, NY USA; 14 Dana-Farber Cancer Institute, Boston, MA USA; 15 Memorial Sloan Kettering Cancer Center, New York, NY USA

**Keywords:** Cancer epidemiology, Epidemiology, Breast cancer

## Abstract

U.S. Black women have disproportionately high breast cancer mortality, partly due to later-stage diagnoses. We examined how social drivers of health (SDOH) relate to stage at diagnosis by analyzing data from 4,995 breast cancer survivors in the Black Women’s Health Study, Carolina Breast Cancer Study, and Women’s Circle of Health Studies. SDOH were self-reported and stage was ascertained from medical records. We used polytomous logistic regression to estimate odds ratios (ORs) for diagnosis at stages III/IV or II versus stage I (referent), adjusting for age, insurance status, and income. Meta-analyzed results indicated that underutilization of screening mammography (OR = 3.21, 95% CI 1.90–5.43) and income below the federal poverty line (OR = 1.91, 95% CI 1.17–3.10) were significantly associated with later stage diagnosis (III/IV). ORs for lack of insurance and lower education were above 1.0, but not consistently statistically significant. These findings substantiate the importance of the affordability and utilization of breast cancer screening.

## Introduction

In the U.S., Black breast cancer patients are 38% more likely to die from their cancer than White breast cancer patients^1,2^. While the higher incidence of triple-negative breast cancer (TNBC) among Black women contributes to the ongoing Black-White disparity in breast cancer survival^3,4^, other factors, including diagnostic delays and the greater frequency of advanced stage at diagnosis among Black versus White patients, also drive survival disparities^5–9^.

Stage at diagnosis is an important predictor of breast cancer prognosis^9^. Past analyses of Surveillance, Epidemiology, and End Results (SEER) Program data have repeatedly indicated that social drivers of health (SDOH), including socioeconomic factors and access to healthcare, are associated with stage at diagnosis. Both a SEER-National Longitudinal Mortality Study linkage (8% Black) and a more recent SEER study (15.9% Black) reported that lower income and living below the federal poverty line are associated with later stage at breast cancer diagnosis, even after controlling for age, time period, and SEER registry^6,10^. These and other SEER-based analyses also reported that women who are uninsured are more likely to be diagnosed with late-stage breast cancer^6,11^.

Regular utilization of screening mammography is another well-documented driver of lower breast cancer stage at diagnosis^12–14^. Both the 2009 and 2016 U.S. Preventive Services Task Force (USPSTF) recommendations for breast cancer screening stated that women ages 40–49 should undergo screening mammograms at the frequency recommended by their health provider, while average-risk women ages 50–74 should obtain biennial screening mammograms^15,16^. Data collected by the CDC have increasingly indicated that Black women are as likely or more likely to engage in screening mammography when compared with women from other racial groups^17–19^; however, as of the most recent CDC report, >17% of Black women aged 50–74 had not obtained a screening mammogram during the previous two years^18^. U.S.-based studies among diverse populations have indicated that underutilization of screening mammography is driven by both structural and individual-level barriers to screening, including financial medical hardship, poverty, lack of insurance, and inconsistent access to medical care^17,20–22^, but more work is needed to understand the barriers most relevant to U.S. Black women.

The goal of this study was to evaluate associations of individual-level SDOH with stage at diagnosis among U.S. Black women. Leveraging data from nearly 5000 Black breast cancer patients enrolled in The Black Women’s Health Study (BWHS), the Carolina Breast Cancer Study Phase 3 (CBCS), and the Women’s Circle of Health (WCHS) and Women’s Circle of Health Follow-up (WCHFS) Studies, we evaluated the potential impact of marital status, education, income, health insurance status, and use of preventive health services on stage at diagnosis. We also evaluated mammography use, a factor strongly influenced by SDOH. Finally, we assessed the extent to which utilization of screening mammography may modify associations between SDOH and stage at diagnosis.

## Results

In total, analyses were completed with data from 4995 women with breast cancer, including 2230 with stage I, 1891 with stage II, 676 with stage III, and 198 with stage IV disease (Table 1). Stage distributions differed by study with the highest proportion of stage I diagnoses observed among BWHS participants and the highest proportion of stage III and IV diagnoses observed among CBCS participants. SDOH distributions also differed by study with the greatest differences observed for years of education and income. In the BWHS, 60.8% of participants had at least 16 years of education in comparison to 35.8% of CBCS participants and 29.6% of WCHS/WCHFS participants. Additionally, only 4.3% of BWHS participants, but 25.6% of CBCS participants and 19.9% of WCHS/WCHFS participants, reported a household income below the federal poverty line.

**Table 1:** Participant characteristics by study*

	BWHS *n* = 1777	CBCS *n* = 1493	WCHS/WCHFS *n* = 1725
Age at diagnosis, *n* (%)			
≥70	339 (19.1)	80 (5.4)	129 (7.5)
60–69	556 (31.9)	288 (19.3)	404 (23.4)
50–59	548 (30.8)	385 (25.8)	565 (32.8)
40–49	296 (16.7)	536 (35.9)	446 (25.9)
<40	28 (1.5)	204 (13.7)	181 (10.5)
Stage at diagnosis, *n* (%)			
I	958 (53.9)	512 (34.3)	760 (44.1)
II	557 (31.3)	658 (44.1)	676 (39.2)
III	187 (10.5)	250 (16.7)	239 (13.9)
IV	75 (4.3)	73 (4.9)	50 (2.9)
ER status, *n* (%)			
ER+	1195 (67.2)	919 (61.6)	1176 (68.2)
ER−	529 (29.8)	499 (33.4)	532 (30.8)
Missing	53 (3.0)	75 (5.0)	17 (1.0)
Marital status, *n* (%)			
Married/living as married	823 (46.3)	619 (41.5)	619 (35.9)
Single	325 (18.3)	295 (19.7)	587 (34.0)
Separated/divorced/widowed	628 (35.3)	578 (38.7)	519 (30.1)
Years of education, *n* (%)			
≥16 years	1080 (60.8)	535 (35.8)	511 (29.6)
13–15 years	460 (25.9)	502 (33.7)	513 (29.7)
≤12 years	235 (13.2)	455 (30.4)	700 (40.6)
Household income^a^, *n* (%)			
High	302 (17.0)	117 (7.8)	287 (16.6)
Medium high	669 (37.6)	292 (19.6)	383 (22.2)
Medium low	515 (29.0)	517 (34.7)	383 (22.2)
Low	190 (10.1)	487 (32.6)	554 (32.1)
Missing	101 (5.7)	80 (5.4)	118 (6.8)
Income for household size^b^, *n* (%)			
Above the federal poverty line	1558 (87.6)	1028 (68.9)	1263 (73.2)
Below the federal poverty line	76 (4.3)	383 (25.6)	343 (19.9)
Missing	143 (8.1)	82 (5.5)	119 (6.9)
Health insurance, *n* (%)			
Yes	1623 (91.3)	1369 (91.7)	1508 (87.4)
No	124 (7.0)	123 (8.2)	217 (12.6)
Regular preventive care^c^, *n* (%)			
Yes	1488 (83.7)	1219 (81.6)	–
No	261 (14.7)	274 (18.4)	–
Adherent to mammography screening guidelines^d^, *n* (%)			
Yes	1473 (82.9)	1070 (71.7)	1377 (79.8)
No	291 (16.4)	375 (25.1)	265 (15.4)

*BWHS* Black Women’s Health Study, *CBCS* Carolina Breast Cancer Study, *WCHS* Women’s Circle of Health Study, *WCHFS* Women’s Circle of Health Follow-up Study, *ER* Estrogen receptor.

*Percentages may not add to 100% due to rounding or missing data. A missing category is displayed for all variables with >5% missing.

^a^Category definitions varied by study. BWHS: >$100,000, $50,001–$100,000, $25,001–$50,000, and ≤$25,000; WCHS/WCHFS: ≥$90,000, $50,000–$89,999, $25,000–$49,999, and <$25,000; CBCS: >$100,000, $50,001–$100,000, $20,000–$50,000, and <$20,000

^b^BWHS and CBCS calculations were based off of the 2010 poverty line. The WCHS/WCHFS calculation was based on the 2013 poverty line.

^c^Preventive care was not queried in WCHS/WCHFS.

^d^BWHS and WCHS/WCHFS defined adherent as under age 50 or age ≥50 with utilization of screening mammography. CBCS defined adherent as under age 45 or age ≥45 with utilization of screening mammography.

Study-specific findings are presented in Supplementary Tables 1–3, and meta-analyzed results are displayed in Table 2. We observed a strong positive association between underutilization of screening mammography and later stage at diagnosis (BWHS OR = 3.03 [1.93–4.74]; CBCS OR = 3.30 [2.25–4.84]; WCHS/WCHFS OR = 3.28 [2.16–4.97]; meta-analysis OR = 3.21 [1.90–5.43] for stage III/IV versus I). Household income, particularly household income below the federal poverty line, was also consistently associated with later stage at diagnosis (BWHS OR = 1.98 [1.06–3.70]; CBCS OR = 2.11 [1.52–2.94]; WCHS/WCHFS OR = 1.69 [1.20–2.37]; meta-analysis OR = 1.91 [1.17–3.10]). Other variables associated with later stage at diagnosis in some, but not all, studies included underutilization of preventive healthcare services other than mammography (BWHS OR = 1.64 [1.14–2.38]; CBCS OR = 1.68 [1.12–2.51]; WCHS/WCHFS=not measured) and lack of health insurance (BWHS OR = 1.30 [0.78–2.12]; CBCS OR = 1.16 [0.67–1.99]; WCHS/WCHFS OR = 1.74 [1.17–2.59]; meta-analysis OR = 1.45 [0.80–2.62]). None of the three studies observed statistically significant associations for marital status or years of education with stage at diagnosis. Enrollment in private health insurance, compared to no insurance, Medicare, or Medicaid, was associated with greater utilization of screening mammography among those eligible for screening (BWHS = not measured; CBCS OR = 2.69 [1.95–3.72]; WCHS/WCHFS OR = 1.45 [0.84–2.49]).

**Table 2:** Meta-analyzed odds ratios comparing odds of breast cancer diagnosis at stage II, or III/IV versus stage I by social drivers of health

	Stage I			Stage II				Stage III/IV			
	BWHS *N*	CBCS *N*	WCHS/ WCHFS *N*	BWHS *N*	CBCS *N*	WCHS/ WCHFS *N*	Meta-analyzed OR^a^ (95% CI)	BWHS *N*	CBCS N	WCHS/WCHFS N	Meta-analyzed OR^a^ (95% CI)
Marital status											
Married/living as married	445	224	284	259	278	243	Reference	119	117	92	Reference
Single	167	94	264	109	127	233	1.02 (0.70, 1.48)	49	74	90	1.02 (0.63, 1.65)
Separated/divorced/widowed	346	194	212	189	253	200	1.07 (0.77, 1.48)	93	131	107	1.21 (0.80, 1.85)
Years of education											
≥16 years	581	167	236	348	201	193	Reference	151	87	82	Reference
13–15 years	249	159	222	145	231	207	1.10 (0.78, 1.55)	66	112	84	1.14 (0.73, 1.78)
≤12 years	126	186	302	64	226	275	1.05 (0.71, 1.55)	45	123	123	1.21 (0.74, 1.97)
Household income^b^											
High	164	42	149	101	61	104	Reference	37	14	34	Reference
Medium high	364	118	185	211	124	142	1.00 (0.64, 1.56)	94	50	56	1.29 (0.68, 2.45)
Medium low	282	186	159	152	223	153	1.09 (0.69, 1.72)	81	108	71	1.66 (0.88, 3.12)
Low	92	145	223	64	211	221	1.37 (0.82, 2.29)	34	131	110	2.17 (1.08, 4.37)
Income for household size^c^											
Above the federal poverty line	846	387	591	488	598	477	Reference	224	193	195	Reference
Below the federal poverty line	32	104	125	28	60	143	1.42 (0.94, 2.14)	16	110	75	1.91 (1.17, 3.10)
Health insurance											
Yes	881	476	686	507	598	587	Reference	235	294	235	Reference
No	64	35	74	36	60	89	1.14 (0.69, 1.88)	24	28	54	1.45 (0.80, 2.62)
Utilized routine screening mammography^d^											
Yes	647	289	408	291	249	260	Reference	133	91	82	Reference
No	60	95	82	43	168	123	1.94 (1.25, 3.00)	39	112	60	3.21 (1.90, 5.43)
Below age for universal screening	250	106	235	222	221	260	1.52 (1.03, 2.25)	88	114	132	1.62 (0.97, 2.71)

*BWHS* Black Women’s Health Study, *CBCS* Carolina Breast Cancer Study, *WCHS* Women’s Circle of Health Study, *WCHFS* Women’s Circle of Health Follow-up Study, *OR* odds ratio, *CI* Confidence Interval.

^a^ORs were adjusted for age at diagnosis (continuous), income for household size, and insurance status across all three studies

^b^Category definitions varied by study. BWHS: >$100,000, $50,001–$100,000, $25,001–$50,000, and ≤$25,000; WCHS/WCHFS: ≥$90,000, $50,000–$89,999, $25,000–$49,999, and <$25,000; CBCS: >$100,000, $50,001–$100,000, $20,000–$50,000, and <$20,000

^c^ BWHS and CBCS calculations were based off of the 2010 poverty line. The WCHS/WCHFS calculation was based on the 2013 poverty line.

^d^ BWHS and WCHS/WCHFS defined age 50 as the age for universal screening, while CBCS used age 45 as the universal screening cutoff.

**Fig. 1:**
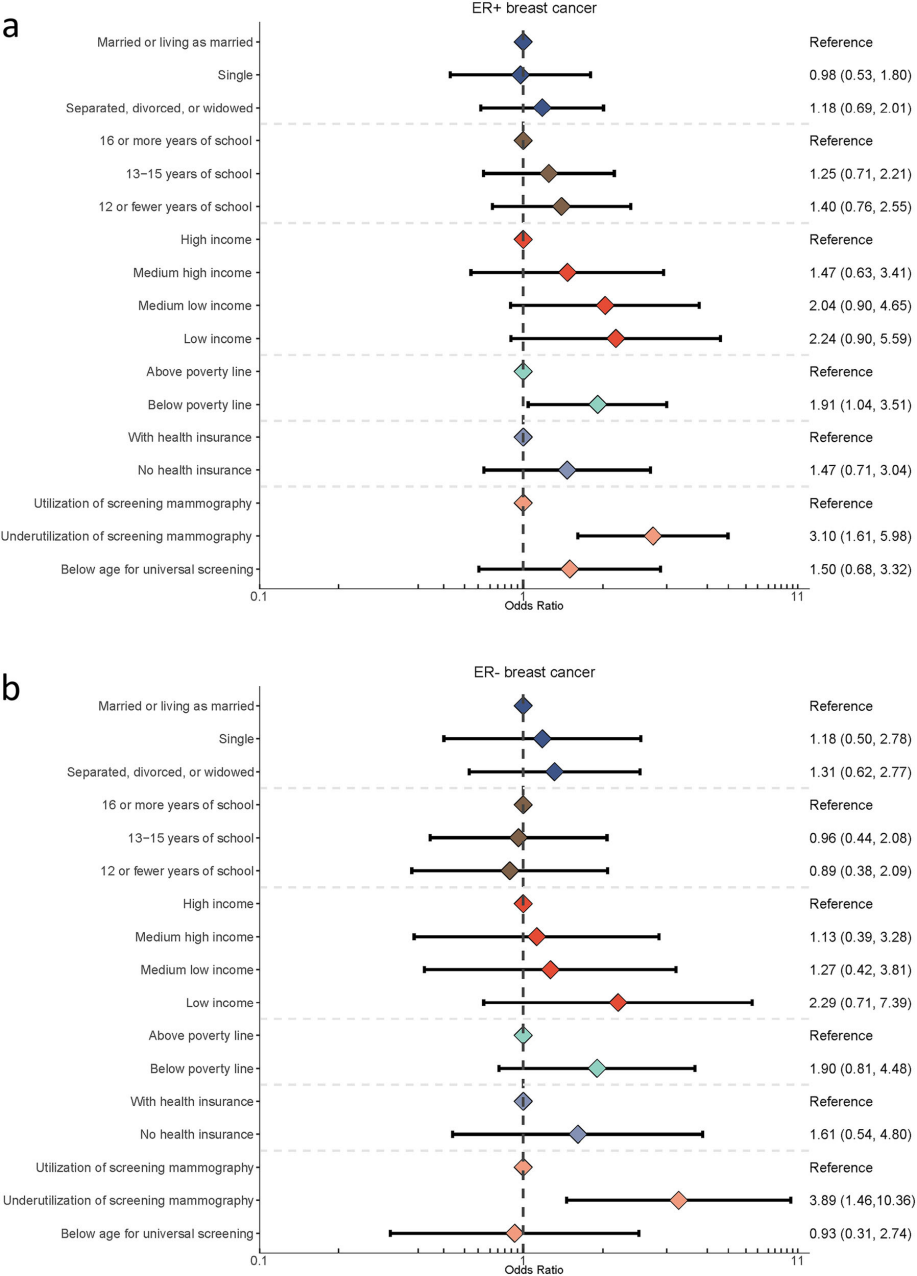
Social drivers of health and stage at diagnosis by ER status. Panels show the odds of breast cancer diagnosis at stage III or stage IV versus stage I by social drivers of health among ER+ breast cancer cases (**a**) and ER- breast cancer cases (**b**).

Results from analyses stratified by tumor ER status varied by study (Supplementary Tables 4–6). When results were combined across studies using meta-analysis, the associations between SDOH and stage at diagnosis were similar across ER+ cases and ER‒ cases (Fig. 1). For example, ORs for underutilization of screening mammography and later stage at diagnosis were 3.10 (1.61, 5.98) for ER+ cases, and 3.89 (1.46, 10.36) for ER‒ cases. Likewise, ORs for household income below the federal poverty line and later stage at diagnosis were 1.91 (1.04, 3.51) for ER+ cases, and 1.90 (0.81, 4.48) for ER‒ cases.

**Fig. 2:**
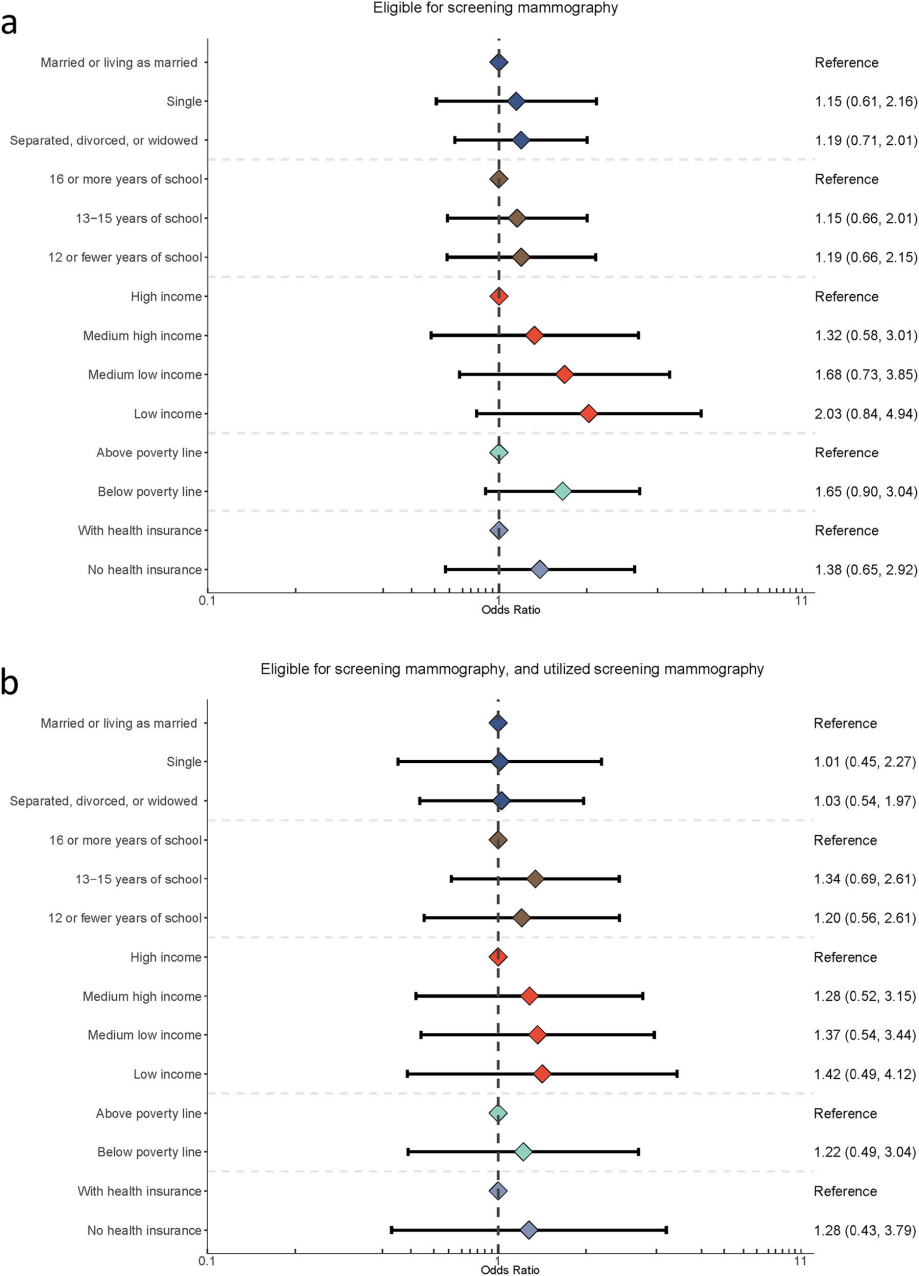
Social drivers of health and stage at diagnosis by mammography utilization. Panels show the odds of breast cancer diagnosis at stage III or stage IV versus stage I by social drivers of health among women old enough to be universally recommended screening mammography (**a**) and among women who utilized screening mammography (**b**).

Associations between SDOH and later stage at diagnosis were either attenuated or remained non-significant in analyses restricted to those who had utilized screening mammography in the years leading up to their diagnosis (Fig. 2, Supplementary Tables 7–9). Meta-analyzed results indicated an OR = 1.65 (0.90, 3.04) for the association between household income below the federal poverty line and later stage at diagnosis among all women eligible for screening mammography, but a comparable OR = 1.22 (0.49, 3.04) among the subset of women who were both eligible for and utilized screening mammography. ORs for lack of health insurance were also attenuated when comparing all screen-eligible women (OR = 1.38 [0.65–2.92]) to the subset of those women who utilized screening mammography (OR = 1.28 [0.43, 3.79]). Associations of marital status and education with later stage at diagnosis were small in magnitude and not statistically significant in either population. Among the two studies with preventive healthcare variables, ORs for preventive health care and late stage at diagnosis attenuated from 1.46 (0.92, 2.30) to 1.25 (0.71, 2.19) in the BWHS, and from 1.75 (1.06 to 2.88) to 0.70 (0.27–1.83) in the CBCS, after restriction to women who accessed screening mammography.

## Discussion

In this study of nearly 5000 U.S. Black women diagnosed with invasive breast cancer, underutilization of screening mammography was strongly associated with later stage at diagnosis. Socioeconomic factors, including low household income, were also associated with later stage at diagnosis, though none of the associations between socioeconomic factors and stage at diagnosis were statistically significant when the analyses were restricted to women who adhered to screening mammography guidelines.

The majority of prior studies on individual-level SDOH and stage at diagnosis used national databases to evaluate how insurance status and screening mammography influence stage at diagnosis. Our findings were generally consistent with findings from these studies, despite the national databases including mostly non-Hispanic White women. With respect to mammography, it is well-established that the primary benefit of screening mammography is the ability to detect breast cancer at an earlier stage, thereby reducing treatment-related burdens and breast cancer mortality^15,16,23–25^. In a study of women aged 49–74 years and living in the Netherlands, women who were not screened had 5.76 (95% CI: 5.47–6.07) times the odds of a stage III/IV diagnosis compared to those who were screened^24^. Therefore, our finding of a 3.21-fold (95% CI:1.90–5.43) increased risk of later stage at diagnosis among those who underutilized screening mammography was not surprising. Prior research has indicated that financial, structural, and personal factors can interfere with adherence to screening guidelines. Financial accessibility is especially important. Despite the Affordable Care Act (ACA) eliminating cost-sharing for screening mammography^26,27^, an analysis of National Health Interview Survey data collected after the ACA went into effect found that 23% of women aged 50-64 and 12% of women aged 65-74 who had a screening mammogram in the past year were charged for part or all of the cost^28^. Women who were uninsured or had Medicare-only coverage were the most likely to have out-of-pocket costs^28^. This is important because other studies have noted that individuals who report paying any proportion of the cost of a screening mammogram or follow-up tests are less likely to return for another screening mammogram within 1–2 years^29^. Those who report “cost as a barrier to healthcare” are also more than twice as likely to not follow screening mammography guidelines^18,30^.

Indirect costs, practical concerns, and psychosocial factors are also important barriers to screening mammography, including among Black women. For example, a qualitative analysis of interview data from 39 Black women who visited an emergency department in Kentucky for non-urgent care highlighted numerous barriers to screening mammography, including lack of transportation, childcare concerns, difficulty navigating the healthcare system, lack of information regarding screening appointments, and personal attitudes toward mammography^31^. These findings align with broader research indicating that individuals experiencing economic instability, food insecurity, social isolation, and lack of access to healthcare are significantly less likely to undergo routine cancer screenings^30,32,33^. Other social risk factors such as younger age, not being married, rural residence, life dissatisfaction, lack of emotional support, and social isolation have also been associated with lower screening adherence^18,30,34^.

Prior research has shown that racial disparities in stage at diagnosis and survival persist in some, but not all, equal-access healthcare systems^35–38^. Therefore, another research priority is to ensure that the recently expanded USPTF breast cancer screening guidelines which advise all women to initiate mammography screening by age 40 are appropriate for Black women^25^. This is important because, on average, Black women are diagnosed with breast cancer at a younger age than their non-Hispanic White counterparts^23,39,40^.

With regard to lack of health insurance, a factor for which we observed a 1.45-fold increased odds of later stage at diagnosis (95% CI: 0.80–2.62), multiple independent analyses of the National Cancer Database have indicated that uninsured and Medicaid-insured patients are 1.5 to 2 times as likely to be diagnosed with stage III or IV breast cancer when compared with privately insured patients^41,42^. An analysis of SEER data also reported associations between being uninsured or underinsured and later stage at diagnosis^11^. The same SEER study additionally conducted a formal mediation analysis to understand the extent to which associations between race and stage at diagnosis are mediated by insurance status. They noted that 45% of the observed association between Black race and advanced stage at diagnosis was mediated by health insurance status, emphasizing that at least a portion of the upstaging seen among Black breast cancer patients could be modified at a policy level^11^. Our finding of attenuated associations between socioeconomic factors and stage at diagnosis among women adherent to screening mammography guidelines further underscores the potential importance of policy-level interventions.

Strengths of our study included the inclusion of nearly 5000 Black women with breast cancer from diverse geographic and socioeconomic backgrounds, and the simultaneous evaluation of multiple, individual-level SDOH. However, it is important to note that these women are not perfectly representative of the full population of US Black women with breast cancer. The BWHS is a prospective cohort of Black women that invited women to participate through postal questionnaires sent to subscribers to *Essence* magazine and members of Black professional organizations^43^. Consistent with this recruitment approach, we observed that BWHS participants with breast cancer had higher income and education than WCHS/WCHFS and CBCS participants. Cases in CBCS and the majority of cases in WCHS/WCHFS were identified through the NC and NJ state cancer registries, respectively, so are more representative of all underlying cancer cases in their regions. However, 13% of WCHS participants were enrolled through a limited number of hospitals in New York City^44^, and the CBCS oversampled cases under age 50^45^. Study limitations included inconsistencies in how some SDOH factors were defined across the three contributing studies, reliance on self-reported data on individual SDOH factors, and imprecision in some estimates (e.g., due to the small number of women who were without health insurance or living in a household with income below the federal poverty line). In conclusion, our study provides evidence that underutilization of mammography screening is a major contributor to later stage at breast cancer diagnosis among Black women. Low household income is also a significant driver of later-stage diagnosis. Policy-level interventions to address barriers to screening adherence, such as eliminating cost-sharing and expanding Medicaid, have shown promise in reducing stage at diagnosis^27,46^. Addressing additional social drivers of health, including access to reliable transportation, financial security, and healthcare accessibility, remains critical for the early detection of breast cancer.

## Methods

### Study population

We included data from self-identified Black women with invasive breast cancer who had previously enrolled in the BWHS, CBCS, or WCHS/WCHFS. Details on the design of each study have been published previously^43,44,47–50^.

### Black Women’s Health Study

The BWHS is a prospective cohort study of 59,000 U.S. Black women who enrolled in the study in 1995 by completing a baseline health and lifestyle questionnaire^43,48^. BWHS participants were ages 21-69 at the time of enrollment and have responded to follow-up questionnaires every two years since, with SDOH first queried in detail in 2003. BWHS cancer diagnoses are identified through self-report on biennial questionnaires with additional details, including stage, obtained via medical record review and linkage to state cancer registries. As of 05/31/2022, 2786 BWHS participants had been diagnosed with invasive breast cancer with a known stage at diagnosis. Of these, 1777 obtained their breast cancer diagnosis after responding to SDOH questions on the 2003 questionnaire and, therefore, were eligible for inclusion in this analysis. The BWHS study protocol was approved by the Boston University Medical Campus Institutional Review Board (IRB), and informed consent was implied by return of the baseline questionnaire.

### Carolina Breast Cancer Study Phase 3

The CBCS is a population-based study that enrolled breast cancer cases ages 23-74 who were living in Eastern or Central North Carolina at the time of their breast cancer diagnosis (2008–2013)^47,50^. The analytic sample included 1495 self-identified Black women. Data on individual-level SDOH at the time of diagnosis and information on suspected and established breast cancer risk factors were collected as part of a home interview that was conducted approximately six months post-diagnosis. Cancer characteristics, including stage at diagnosis, were abstracted from medical records, including breast cancer pathology reports. Of the Black CBCS cases, 1493 had data on at least one SDOH and stage at diagnosis and, therefore, were eligible for inclusion in this analysis. The CBCS study protocol was approved by the IRB at the University of North Carolina Chapel Hill School of Medicine. Written informed consent was provided by each participant.

### Women’s Circle of Health Study and the Women’s Circle of Health Follow-up Study

The WCHS is a multi-site case-control study including breast cancer cases who self-identified as Black or African American, diagnosed at ages 20–75 in New York City and New Jersey from 2001 to 2013^44^. The study continued as the WCHFS, enrolling Black women in New Jersey diagnosed from 2013 to 2019, with ongoing follow-up^49^. Overall, 1790 Black women with invasive breast cancer were enrolled in the studies. Demographic data, health and lifestyle data, and data on individual-level SDOH at the time of diagnosis were collected as part of a home interview conducted approximately ten months after diagnosis. Cancer characteristics, including stage at diagnosis, were obtained through medical record review, pathology report review, and linkage to state cancer registries. A total of 1725 cases had data on at least one SDOH and stage at diagnosis and, therefore, were eligible for inclusion in this analysis. The study protocol was approved by the IRBs of all participating institutions, including Roswell Park Comprehensive Cancer Center and Rutgers University. Written informed consent was obtained from each participant.

### Exposure and covariate assessment

We included SDOH that had been queried as part of at least two of the participating studies. Marital status was queried by all three studies and harmonized to a three-level variable (married/living as married, single, separated/widowed/divorced). Number of years of education was also queried across all three studies and harmonized to a three-level variable (≤12 years, 13–15 years, ≥16 years). Household income was queried by all three studies (BWHS: >$100,000, $50,001-$100,000, $25,001–$50,000, and ≤$25,000; WCHS/WCHFS: ≥$90,000, $50,000-$89,999, $25,000–$49,999, and <$25,000; CBCS: >$100,000, $50,001–$100,000, $20,000–$50,000, and <$20,000) and harmonized to a four-level variable (high income, medium-high income, medium-low income, and low income). This variable was combined with information on household size to derive an additional two-level variable denoting whether the income for household size was above or below the federal poverty line. Also available across all three studies was health insurance status (yes, no). More detailed health insurance information (private versus Medicare or Medicaid) was only available in the CBCS and WCHS/WCHFS. Information on utilization of routine screening mammography (yes, no, below age for universally-recommended screening) was available for all three studies. The age cutoff for “below age for universally-recommended screening” was 50 years for BWHS, 50 years for WCHS/WCHFS, and 45 years for CBCS due to differences in how mammography screening data were collected. Consistent with the median years of diagnosis in our study populations, the cutoffs for all studies were informed by the 2009 and 2016 USPTF screening guidelines. These guidelines advised an individualized decision to start screening mammography for women 40-50 years of age and universal screening mammography starting at age 50^15,16,51^. Information on regular uptake of other preventive care measures was only available in the BWHS (i.e., self-report of at least two of the following in the 2–4 years prior to diagnosis: annual physical, pap smear, blood sugar test) and CBCS (i.e., self-report of usually accessing medical care through a general practitioner or specialist during the 10 years prior to diagnosis). Age at diagnosis (continuous covariate) was available for all study participants.

### Outcome assessment

Data on stage at breast cancer diagnosis were obtained via medical record review or linkage to a state cancer registry. Two categories of advanced stage (stage II; stage III or IV) were compared to stage I. Stages III and IV were combined due to the small number of stage IV participants in each study (BWHS *n* = 75, CBCS *n* = 73, WCHS/WCHFS *n* = 50).

### Statistical analysis

We used polytomous logistic regression adjusted for age at diagnosis, insurance status, and household income below the federal poverty line to estimate study-specific odds ratios (OR) and 95% confidence intervals (CI) for the associations between each categorical SDOH exposure and stage at diagnosis. We additionally estimated associations between SDOH and stage at diagnosis among subpopulations defined by (1) breast cancer estrogen receptor (ER) status and (2) utilization of screening mammography. For SDOH measured across all three studies, estimates were combined across studies using fixed effects meta-analysis of log-scale odds ratios. Study-specific analyses were completed using SAS version 9.4 (Cary, NC) and R version 4.4.2, and meta-analyses were completed in R version 4.4.3 using the metafor package.

## Supplementary information

Supplementary Information

## Data Availability

To protect the privacy of individuals who participated in the BWHS, WCHS/WCHFS, and CBCS, the data underlying this article cannot be shared on a publicly accessible database. The data can be shared with individual investigators upon approval from the PIs of each study and with appropriate IRB approval and data transfer agreements. For more information contact the corresponding author.

## Code availability

The code needed to replicate the present analyses is stored at Boston University, Rutgers University, the University of North Carolina, and Thomas Jefferson University, and can be shared on reasonable request to the corresponding author.
