# Supplementary material for: Associations between social drivers of health and breast cancer stage at diagnosis among U.S. Black women
Source: NPJ Breast Cancer. 2025 Aug 6;11:85. doi: 10.1038/s41523-025-00804-0 (PMC12328792; doi:10.1038/s41523-025-00804-0)
Supplement: Supplementary file 1 — Supplementary Information [file 41523_2025_804_MOESM1_ESM.pdf]

Supplemental Table 1. Multivariable-adjusted odds ratios comparing odds of breast cancer diagnosis at stage II or III/IV versus stage I by social drivers of health in the Black Women's Health Study

	Stage I (n=958)	Stage II (n=557)		Stage III/IV (n=262)	
	N (%)	N (%)	MV OR[a] (95%CI)	N (%)	MV OR[a] (95%CI)
Marital status					
Married/living as married	445 (46.5)	259 (46.5)	Reference	119 (45.6)	Reference
Single	167 (17.4)	109 (19.6)	0.98 (0.74, 1.32)	49 (18.8)	0.90 (0.62, 1.34)
Separated/divorced/widowed	346 (36.1)	189 (33.9)	1.04 (0.82, 1.32)	93 (35.6)	1.12 (0.82, 1.54)
Years of education					
≥16 years	581 (60.8)	348 (62.5)	Reference	151 (57.6)	Reference
13-15 years	249 (26.1)	145 (26.0)	1.00 (0.78, 1.28)	66 (25.2)	1.06 (0.76, 1.48)
≤12 years	126 (13.2)	64 (11.5)	0.88 (0.62, 1.24)	45 (17.2)	1.46 (0.98, 2.18)
Household income					
>$100,000	164 (17.1)	101 (18.1)	Reference	37 (14.1)	Reference
$50,001-$100,000	364 (38.0)	211 (37.9)	0.98 (0.72, 1.32)	94 (35.9)	1.19 (0.78, 1.83)
$25,001-$50,000	282 (29.4)	152 (27.3)	0.94 (0.68, 1.29)	81 (30.9)	1.36 (0.88, 2.11)
≤$25,000	92 (9.6)	64 (11.5)	1.26 (0.83, 1.90)	34 (13.0)	1.83 (1.06, 3.14)
Income for household size[b]					
Above the federal poverty line	846 (88.3)	488 (87.6)	Reference	224 (85.5)	Reference
Below the federal poverty line	32 (3.3)	28 (5.0)	1.62 (0.96, 2.72)	16 (6.1)	1.98 (1.06, 3.70)
Health insurance					
Yes	881 (92)	507 (91.0)	Reference	235 (89.7)	Reference
No	64 (6.7)	36 (6.5)	0.92 (0.60, 1.40)	24 (9.2)	1.30 (0.78, 2.12)
Regular preventive care[c]					
Yes	825 (86.1)	453 (81.3)	Reference	210 (80.2)	Reference
No	118 (12.3)	92 (16.5)	1.42 (1.06, 1.92)	51 (19.5)	1.64 (1.14, 2.38)
Utilized routine screening mammography					
Age ≥50: Yes	647 (67.5)	291 (52.2)	Reference	133 (50.8)	Reference
Age ≥50: No[d]	60 (6.3)	43 (7.72)	1.56 (1.03, 2.37)	39 (14.9)	3.03 (1.93, 4.74)
Age<50	250 (26.1)	222 (39.9)	1.95 (1.55, 2.45)	88 (33.6)	1.73 (1.27, 2.35)

[a] ORs were adjusted for age at diagnosis (continuous), income for household size, and insurance status.

[b] Calculation was based off of the 2010 poverty line.

[c] Receipt of regular preventive care was defined as at least two of the following in the questionnaire cycle prior to diagnosis: physical exam, pap smear, blood sugar test.

[d] "No" was defined as: no report of a screening mammogram during the 2-4-year period prior to diagnosis among those 50 or older during the 2-4-year period prior to diagnosis.

Supplemental Table 2. Multivariable-adjusted odds ratios comparing odds of breast cancer diagnosis at stage II or III/IV versus stage I by social drivers of health in the Carolina Breast Cancer Study

	Stage I (n=512)	Stage II (n=658)		Stage III/IV (n=323)	
	N (%)	N (%)	MV OR[a] (95%CI)	N (%)	MV OR[a] (95%CI)
Marital status					
Married/living as married	224 (43.8)	278 (42.3)	Reference	117 (36.3)	Reference
Single	94 (18.4)	127 (19.3)	0.88 (0.62, 1.24)	74 (23.0)	1.01 (0.67, 1.52)
Separated/divorced/widowed	194 (37.9)	253 (38.5)	1.21 (0.92, 1.61)	131 (40.7)	1.28 (0.91, 1.81)
Years of education					
≥16 years	167 (32.6)	201 (30.6)	Reference	87 (27.0)	Reference
13-15 years	159 (31.1)	231 (35.1)	1.28 (0.95, 1.73)	112 (34.8)	1.31 (0.91, 1.91)
≤12 years	186 (36.3)	226 (34.4)	1.05 (0.77, 1.45)	123 (38.2)	1.12 (0.76, 1.66)
Household income					
>$100,000	42 (8.6)	61 (9.9)	Reference	14 (4.6)	Reference
$50,001-$100,000	118 (24.0)	124 (20.0)	0.79 (0.49, 1.27)	50 (16.5)	1.37 (0.68, 2.76)
$20,000-$50,000	186 (37.9)	223 (36.0)	0.92 (0.58, 1.44)	108 (35.6)	1.93 (1.00, 3.74)
<$20,000	145 (29.5)	211 (34.1)	1.17 (0.67, 2.04)	131 (43.2)	2.97 (1.38, 6.39)
Income for household size[b]					
Above the federal poverty line	387 (78.8)	598 (90.9)	Reference	193 (63.7)	Reference
Below the federal poverty line	104 (21.2)	60 (9.1)	1.38 (1.04, 1.85)	110 (36.3)	2.11 (1.52, 2.94)
Health insurance					
Yes	476 (93.2)	598 (90.9)	Reference	294 (91.3)	Reference
No	35 (6.9)	60 (9.1)	1.30 (0.82, 2.06)	28 (8.7)	1.16 (0.67, 1.99)
Regular preventive care[c]					
Yes	447 (87.3)	529 (80.4)	Reference	243 (75.2)	Reference
No	65 (12.7)	129 (19.6)	1.39 (0.98, 1.99)	80 (24.8)	1.68 (1.12, 2.51)
Utilized routine screening mammography					
Age ≥45: Yes	289 (59.0)	249 (39.0)	Reference	91 (28.7)	Reference
Age ≥45: No[d]	95 (19.4)	168 (26.3)	1.89 (1.37, 2.60)	112 (35.3)	3.30 (2.25, 4.84)
Age<45	106 (21.6)	221 (34.6)	1.15 (0.76, 1.76)	114 (36.0)	1.40 (0.83, 2.35)

[a] ORs were adjusted for age at diagnosis (continuous), income for household size, and insurance status.

[b] Calculation was based off of the 2010 poverty line.

[c] Receipt of regular preventive care was defined as self-report of usually seeing primary or specialty care doctors for their health needs in the 10 years prior to diagnosis

[d] "No" was defined as fewer than 0.5 mammograms/year

Supplemental Table 3. Multivariable-adjusted odds ratios comparing odds of breast cancer diagnosis at stage II or III/IV versus stage I by social drivers of health in the Women's Circle of Health Study

	Stage I (n=760)	Stage II (n=676)		Stage III/IV (n=289)	
	N (%)	N (%)	MV OR[a] (95%CI)	N (%)	MV OR[a] (95%CI)
Marital status					
Married/living as married	284 (37.4)	243 (35.9)	Reference	92 (31.8)	Reference
Single	264 (34.7)	233 (34.5)	1.15 (0.89, 1.50)	90 (31.1)	1.14 (0.80, 1.62)
Separated/divorced/widowed	212 (27.9)	200 (29.6)	0.99 (0.76, 1.29)	107 (37.0)	1.27 (0.90, 1.79)
Years of education					
≥16 years	236 (31.1)	193 (28.5)	Reference	82 (28.4)	Reference
13-15 years	222 (29.2)	207 (30.6)	1.09 (0.84, 1.43)	84 (29.1)	1.10 (0.78, 1.57)
≤12 years	302 (39.7)	275 (40.7)	1.17 (0.89, 1.53)	123 (42.6)	1.10 (0.76, 1.58)
Household income					
≥$90,000	149 (19.6)	104 (15.4)	Reference	34 (11.8)	Reference
$50,000-$89,999	185 (24.3)	142 (21.0)	1.15 (0.82, 1.61)	56 (19.4)	1.40 (0.86, 2.26)
$25,000-$49,999	159 (20.9)	153 (22.6)	1.41 (1.01, 1.98)	71 (24.6)	1.93 (1.20, 3.10)
<$25,000	223 (29.3)	221 (32.7)	1.53 (1.10, 2.11)	110 (38.1)	2.20 (1.40, 3.48)
Income for household size[b]					
Above the federal poverty line	591 (77.8)	477 (70.6)	Reference	195 (67.5)	Reference
Below the federal poverty line	125 (16.4)	143 (21.1)	1.40 (1.06, 1.85)	75 (25.9)	1.69 (1.20, 2.37)
Health insurance					
Yes	686 (90.3)	587 (86.8)	Reference	235 (81.3)	Reference
No	74 (9.74)	89 (13.2)	1.21 (0.86, 1.69)	54 (18.7)	1.74 (1.17, 2.59)
Regular preventive care[c]					
Yes	-	-	Reference	-	Reference
No	-	-	-	-	-
Utilized routine screening mammography					
Age ≥50: Yes	408 (53.7)	260 (38.5)	Reference	82 (28.4)	Reference
Age ≥50: No[d]	82 (10.8)	123 (18.2)	2.26 (1.63, 3.12)	60 (20.8)	3.28 (2.16, 4.97)
Age<50	235 (30.9)	260 (38.5)	0.96 (0.65, 1.40)	132 (45.7)	1.56 (0.94, 2.60)

[a] ORs were adjusted for age at diagnosis (continuous), income for household size, and insurance status.

[b] Calculation was based off of the 2013 poverty line.

[c] Receipt of regular preventive care prior to breast cancer diagnosis was not queried in the Women's Circle of Health Study.

[d] "No" was defined as: the last screening mammogram was performed more than two years prior to diagnosis.

Supplemental Table 4. Multivariable-adjusted odds ratios comparing odds of breast cancer diagnosis at stage III or IV versus stage I by social drivers of health in the Black Women's Health Study, by ER-status

	ER+ (n=1,195)			ER- (n=529)		
	Stage I (n=701)	Stage III/IV (n=148)		Stage I (n=234)	Stage III/IV (n=100)	
	N (%)	N (%)	MV OR[a] (95%CI)		N (%)	MV OR[a] (95%CI)
Marital status						
Married/living as married	333 (47.5)	69 (46.6)	Reference	100 (42.7)	40 (40.4)	Reference
Single	116 (16.5)	29 (19.6)	1.05 (0.64, 1.71)	49 (20.9)	20 (20.2)	0.74 (0.37, 1.45)
Separated/divorced/widowed	252 (36.0)	50 (33.8)	1.05 (0.70, 1.59)	85 (36.3)	39 (39.4)	1.36 (0.78, 2.36)
Years of education						
≥16 years	429 (61.4)	82 (55.4)	Reference	139 (59.4)	59 (59.0)	Reference
13-15 years	182 (26.0)	37 (25.0)	1.10 (0.71, 1.68)	60 (25.6)	27 (27.0)	1.08 (0.62, 1.90)
≤12 years	88 (12.6)	29 (19.6)	1.79 (1.08, 2.96)	35 (15.0)	14 (14.0)	1.00 (0.48, 2.10)
Household income						
>$100,000	125 (17.8)	17 (11.5)	Reference	37 (15.8)	16 (16.0)	Reference
$50,001-$100,000	264 (37.7)	56 (37.8)	1.66 (0.92, 2.97)	89 (38.0)	31 (31.0)	0.76 (0.37, 1.57)
$25,001-$50,000	198 (28.3)	51 (34.5)	2.01 (1.11, 3.67)	77 (32.9)	29 (29.0)	0.90 (0.43, 1.87)
≤$25,000	71 (10.1)	16 (10.8)	1.89 (0.89, 4.02)	20 (8.6)	16 (16.0)	1.94 (0.78, 4.80)
Income for household size[b]						
Above the federal poverty line	616 (87.9)	128 (86.5)	Reference	210 (89.7)	83 (83.0)	Reference
Below the federal poverty line	22 (3.1)	10 (6.8)	2.30 (1.06, 5.02)	9 (3.8)	6 (6.0)	1.84 (0.61, 5.50)
Health insurance						
Yes	646 (92.2)	132 (89.2)	Reference	214 (91.5)	90 (90.0)	Reference
No	46 (6.6)	13 (8.8)	1.29 (0.68, 2.47)	16 (6.8)	10 (10.0)	1.31 (0.56, 3.07)
Regular preventive care[c]						
Yes	609 (86.9)	116 (78.4)	Reference	198 (84.6)	82 (82.0)	Reference
No	81 (11.6)	31 (21.0)	1.92 (1.20, 3.08)	32 (13.7)	18 (18.0)	1.42 (0.74, 2.73)
Utilized routine screening mammography						
Age ≥50: Yes	483 (68.9)	77 (52.0)	Reference	152 (65.0)	48 (48.0)	Reference
Age ≥50: No[d]	42 (6.0)	21 (14.2)	3.10 (1.72, 5.59)	17 (7.3)	15 (15.0)	2.79 (1.26, 6.15)
Age<50	176 (25.1)	49 (33.1)	1.10 (0.61, 1.97)	64 (27.4)	36 (36.0)	0.74 (0.33, 1.65)

[a] ORs were adjusted for age at diagnosis (continuous), income for household size, and insurance status.

[b] Calculation was based off of the 2010 poverty line.

[c] Receipt of regular preventive care was defined as at least two of the following in the questionnaire cycle prior to diagnosis: physical exam, pap smear, blood sugar test.

[d] "No" was defined as: no report of a screening mammogram during the 2-4-year period prior to diagnosis among those 50 or older during the 2-4-year period prior to diagnosis.

Supplemental Table 5. Multivariable-adjusted odds ratios comparing odds of breast cancer diagnosis at stage III or IV versus stage I by social drivers of health in the Carolina Breast Cancer Study, by ER-status

	ER+ (N=919)			ER- (N=569)		
	Stage I (n=371)	Stage III/IV (n=190)		Stage I (n=140)	Stage III/IV (n=132)	
	N (%)	N (%)	MV OR[a] (95%CI)		N (%)	MV OR[a] (95%CI)
Marital status						
Married/living as married	159 (42.9)	66 (34.9)	Reference	65 (46.4)	50 (37.9)	Reference
Single	77 (20.8)	39 (20.6)	0.74 (0.44, 1.26)	17 (12.1)	35 (26.5)	2.06 (1.00, 4.24)
Separated/divorced/widowed	135 (36.4)	84 (44.4)	1.36 (0.88, 2.11)	58 (41.4)	47 (35.6)	1.13 (0.63, 2.00)
Years of education						
≥16 years	125 (33.7)	36 (27.3)	Reference	41 (29.3)	36 (27.3)	Reference
13-15 years	112 (30.2)	48 (36.4)	1.41 (0.88, 2.26)	47 (33.6)	48 (36.4)	1.12 (0.60, 2.09)
≤12 years	134 (36.1)	48 (36.4)	1.24 (0.76, 2.05)	52 (37.1)	48 (36.4)	0.86 (0.45, 1.67)
Household income						
>$100,000	28 (7.9)	9 (5.1)	Reference	14 (10.4)	5 (4.0)	Reference
$50,001-$100,000	82 (23.0)	26 (14.6)	1.06 (0.44, 2.55)	35 (26.1)	23 (18.6)	2.02 (0.63, 6.43)
$20,000-$50,000	138 (38.8)	58 (32.6)	1.44 (0.63, 3.30)	48 (35.8)	50 (40.3)	3.24 (1.07, 9.81)
<$20,000	108 (30.3)	85 (47.8)	2.26 (0.87, 5.85)	37 (27.6)	46 (37.1)	5.38 (1.44, 20.10)
Income for household size[b]						
Above the federal poverty line	283 (79.5)	107 (60.1)	Reference	103 (76.9)	85 (68.6)	Reference
Below the federal poverty line	73 (20.5)	71 (39.9)	2.41 (1.60, 3.63)	31 (23.1)	39 (31.5)	1.56 (0.88, 2.76)
Health insurance						
Yes	340 (91.9)	171 (90.5)	Reference	135 (96.4)	122 (92.4)	Reference
No	30 (8.1)	18 (9.5)	1.06 (0.55, 2.02)	5 (3.6)	10 (7.6)	1.90 (0.62, 5.86)
Regular preventive care[c]						
Yes	321 (86.5)	141 (74.2)	Reference	125 (89.3)	101 (76.5)	Reference
No	50 (13.5)	49 (25.8)	1.62 (0.99, 2.66)	15 (10.7)	31 (23.5)	1.99 (0.96, 4.12)
Utilized routine screening mammography						
Age ≥45: Yes	208 (58.3)	52 (27.5)	Reference	81 (61.4)	39 (30.7)	Reference
Age ≥45: No[d]	77 (21.6)	70 (37.0)	3.30 (2.05, 5.30)	18 (13.6)	42 (33.1)	4.18 (2.07, 8.42)
Age<45	72 (20.2)	67 (35.5)	1.51 (0.77, 2.93)	33 (25.0)	46 (36.2)	1.45 (0.61, 3.44)

[a] ORs were adjusted for age at diagnosis (continuous), income for household size, and insurance status.

[b] Calculation was based off of the 2010 poverty line.

[c] Receipt of regular preventive care was defined as self-report of usually seeing primary or specialty care doctors for their health needs in the 10 years prior to diagnosis

[d] "No" was defined as fewer than 0.5 mammograms/year

Supplemental Table 6. Multivariable-adjusted odds ratios comparing odds of breast cancer diagnosis at stage III or IV versus stage I by social drivers of health in the Women's Circle of Health studies, by ER-status

	ER+ (N=1,176)			ER- (N=532)		
	Stage I (n=564)	Stage III/IV (n=187)		Stage I (n=188)	Stage III/IV (n=95)	
	N (%)	N (%)	MV OR[a] (95%CI)	N (%)	N (%)	MV OR[a] (95%CI)
Marital status						
Married/living as married	137 (32.9)	158 (43.5)	Reference	77 (41.0)	29 (30.5)	Reference
Single	114 (27.3)	98 (27.0)	1.12 (0.72,1.74)	66 (35.1)	32 (33.7)	1.15 (0.61,2.17)
Separated/divorced/widowed	166 (39.8)	107 (29.5)	1.17 (0.77,1.78)	45 (23.9)	34 (35.8)	1.51 (0.80,2.88)
Years of education						
≥16 years	178 (31.6)	50 (26.7)	Reference	53 (28.2)	30 (31.6)	Reference
13-15 years	158 (28.0)	55 (29.4)	1.29 (0.82,2.02)	64 (34.0)	26 (27.4)	0.69 (0.36,1.33)
≤12 years	228 (40.4)	82 (43.8)	1.27 (0.82,1.96)	71 (37.8)	39 (41.0)	0.84 (0.44,1.58)
Household income						
≥$90,000	108 (19.2)	19 (10.2)	Reference	40 (21.3)	14 (14.7)	Reference
$50,000-$89,999	137 (24.3)	35 (18.7)	1.51 (0.81,2.80)	47 (25.0)	21 (22.1)	1.38 (0.62,3.08)
$25,000-$49,999	119 (21.1)	53 (28.3)	2.48 (1.37,4.49)	37 (19.7)	15 (15.8)	1.15 (0.48,2.73)
<$25,000	167 (29.6)	70 (37.4)	2.48 (1.38,4.46)	53 (28.2)	38 (40.0)	1.93 (0.90,4.14)
Income for household size[b]						
Above the federal poverty line	438 (77.7)	133 (71.1)	Reference	147 (78.2)	57 (60.0)	Reference
Below the federal poverty line	93 (16.5)	43 (23.0)	1.41 (0.92,2.16)	30 (16.0)	31 (32.6)	2.41 (1.31,4.43)
Health insurance						
Yes	510 (90.4)	152 (81.3)	Reference	170 (90.4)	76 (80.0)	Reference
No	54 (9.6)	35 (18.7)	1.88 (1.16,3.03)	18 (9.6)	19 (20.0)	1.75 (0.84,3.63)
Regular preventive care[c]						
Yes	-	-	-	-	-	-
No	-	-	-	-	-	-
Utilized routine screening mammography						
Age ≥50: Yes	308 (54.6)	50 (26.7)	Reference	97 (51.6)	30 (31.6)	Reference
Age ≥50: No[d]	69 (12.2)	36 (19.3)	2.89 (1.74,4.81)	12 (6.4)	21 (22.1)	5.11 (2.20,11.9)
Age<50	165 (29.3)	90 (48.1)	2.14 (1.14,4.03)	67 (35.6)	40 (42.1)	0.76 (0.31,1.89)

[a] ORs were adjusted for age at diagnosis (continuous), income for household size, and insurance status.

[b] Calculation was based off of the 2013 poverty line.

[c] Receipt of regular preventive care prior to breast cancer diagnosis was not queried in the Women's Circle of Health studies.

[d] "No" was defined as: the last screening mammogram was performed more than two years prior to diagnosis.

Supplemental Table 7. Multivariable-adjusted odds ratios comparing odds of breast cancer diagnosis at stage III or IV versus stage I by social drivers of health in the Black Women's Health Study, among screen-eligible women

	Screen-eligible women[a] (N=1,217)			Screen-eligible women[a] who used mammography screening (N=1,071)		
	Stage I (n=708)	Stage III/IV (n=174)		Stage I (n=647)	Stage III/IV (n=133)	
	N (%)	N (%)	MV OR[b] (95%CI)		N (%)	MV OR[b] (95%CI)
Marital status						
Married/living as married	313 (44.2)	36 (20.7)	Reference	291 (45.0)	63 (47.4)	Reference
Single	89 (12.6)	79 (45.4)	0.84 (0.48, 1.46)	79 (12.2)	15 (11.3)	0.76 (0.40, 1.42)
Separated/divorced/widowed	306 (43.2)	59 (33.9)	1.04 (0.72, 1.50)	277 (42.8)	55 (41.4)	0.98 (0.64, 1.46)
Years of education						
≥16 years	405 (57.4)	88 (50.6)	Reference	375 (58.0)	69 (51.9)	Reference
13-15 years	193 (27.3)	50 (28.7)	1.18 (0.80, 1.74)	177 (27.4)	39 (29.3)	1.18 (0.76, 1.82)
≤12 years	108 (15.3)	36 (20.7)	1.48 (0.94, 2.34)	94 (14.6)	25 (18.8)	1.46 (0.86, 2.48)
Household income						
>$100,000	113 (16.0)	22 (12.6)	Reference	108 (16.7)	20 (15.0)	Reference
$50,001-$100,000	259 (36.6)	59 (33.9)	1.23 (0.72, 2.11)	237 (36.6)	50 (37.6)	1.21 (0.68, 2.14)
$25,001-$50,000	217 (30.6)	56 (32.3)	1.42 (0.82, 2.46)	198 (30.6)	41 (30.8)	1.22 (0.68, 2.21)
≤$25,000	75 (10.6)	22 (12.6)	1.65 (0.85, 3.23)	64 (9.9)	13 (9.8)	1.23 (0.57, 2.67)
Income for household size[c]						
Above the federal poverty line	620 (87.6)	142 (81.6)	Reference	572 (88.4)	113 (85.0)	Reference
Below the federal poverty line	24 (3.4)	13 (7.5)	2.40 (1.18, 4.88)	19 (2.9)	7 (5.3)	1.96 (0.80, 4.80)
Health insurance						
Yes	655 (92.5)	157 (90.2)	Reference	605 (93.5)	121 (91.0)	Reference
No	49 (6.9)	15 (8.6)	1.12 (0.60, 2.06)	38 (5.9)	10 (7.5)	1.16 (0.56, 2.42)
Regular preventive care[d]						
Yes	615 (86.9)	143 (82.2)	Reference	571 (88.3)	115 (86.5)	Reference
No	89 (12.6)	31 (17.8)	1.46 (0.92, 2.30)	72 (11.1)	18 (13.5)	1.25 (0.71, 2.19)

[a] Screening eligibility was defined as 50 or older during the 2-4-year period prior to diagnosis.

[b] ORs were adjusted for age at diagnosis (continuous), income for household size, and insurance status.

[c] Calculation was based off of the 2010 poverty line.

[d] Receipt of regular preventive care was defined as at least two of the following in the questionnaire cycle prior to diagnosis: physical exam, pap smear, blood sugar test.

Supplemental Table 8. Multivariable-adjusted odds ratios comparing odds of breast cancer diagnosis at stage III or IV versus stage I by social drivers of health in the Carolina Breast Cancer Study, among screen-eligible women

	Screen-eligible women[a] (N=1,004)			Screen-eligible women[a] who used mammography screening (N=629)		
	Stage I (n=384)	Stage III/IV (n=203)		Stage I (n=289)	Stage III/IV (n=91)	
	N (%)	N (%)	MV OR[b] (95%CI)		N (%)	MV OR[b] (95%CI)
Marital status						
Married/living as married	171 (44.5)	74 (36.5)	Reference	139 (48.1)	37 (40.7)	Reference
Single	53 (13.8)	35 (17.2)	1.25 (0.73, 2.13)	32 (11.1)	13 (14.3)	1.38 (0.65, 2.93)
Separated/divorced/widowed	160 (41.7)	94 (46.3)	1.28 (0.86, 1.92)	118 (40.8)	41 (45.1)	1.24 (0.72, 2.16)
Years of education						
≥16 years	115 (29.9)	52 (25.6)	Reference	95 (32.9)	28 (30.8)	Reference
13-15 years	119 (31.0)	68 (33.5)	1.20 (0.76, 1.89)	97 (33.6)	39 (42.9)	1.39 (0.78, 2.49)
≤12 years	150 (39.1)	83 (40.9)	1.02 (0.64, 1.62)	97 (33.6)	24 (26.4)	0.81 (0.41, 1.59)
Household income						
>$100,000	28 (7.3)	7 (3.4)	Reference	24 (8.3)	6 (6.6)	Reference
$50,001-$100,000	86 (22.4)	31 (15.3)	1.56 (0.61, 3.96)	72 (24.9)	23 (25.3)	1.32 (0.48, 3.64)
$20,000-$50,000	139 (36.2)	73 (36.0)	2.26 (0.93, 5.47)	107 (37.0)	32 (35.2)	1.26 (0.47, 3.36)
<$20,000	115 (29.9)	80 (39.4)	2.91 (1.10, 7.70)	75 (26.0)	24 (26.4)	1.52 (0.47, 4.97)
Income for household size[c]						
Above the federal poverty line	293 (76.3)	131 (64.5)	Reference	228 (78.9)	68 (74.7)	Reference
Below the federal poverty line	75 (19.5)	60 (29.6)	1.73 (1.15, 2.60)	50 (17.3)	17 (18.7)	1.20 (0.64, 2.25)
Health insurance						
Yes	358 (93.2)	181 (89.2)	Reference	275 (95.2)	87 (95.6)	Reference
No	26 (6.8)	22 (10.8)	1.51 (0.80, 2.83)	14 (4.8)	4 (4.4)	0.89 (0.27, 2.93)
Regular preventive care[d]						
Yes	340 (88.5)	157 (77.3)	Reference	264 (91.3)	85 (93.4)	Reference
No	44 (11.5)	46 (22.7)	1.75 (1.06, 2.88)	25 (8.7)	6 (6.6)	0.70 (0.27, 1.83)

[a] Screening eligibility was defined as 45 or older.
[b] ORs were adjusted for age at diagnosis (continuous), income for household size, and insurance status.
[c] Receipt of regular preventive care was defined as self-report of usually seeing primary or specialty care doctors for their health needs in the 10 years prior to diagnosis
[d] "No" was defined as fewer than 0.5 mammograms/year

Supplemental Table 9. Multivariable-adjusted odds ratios comparing odds of breast cancer diagnosis at stage III or IV versus stage I by social drivers of health in the Women's Circle of Health studies, among screen-eligible women

	Screen-eligible women[a] (N=1,098)			Screen-eligible women[a] who used mammography screening (N=750)		
	Stage I (n=525)	Stage III/IV (n=157)		Stage I (n=408)	Stage III/IV (n=82)	
	N (%)	N (%)	MV OR[b] (95%CI)		N (%)	MV OR[b] (95%CI)
Marital status						
Married/living as married	190 (36.2)	45 (28.7)	Reference	160 (39.2)	31 (37.8)	Reference
Single	220 (41.9)	70 (44.6)	1.31 (0.85,2.03)	163 (39.9)	33 (40.2)	1.07 (0.61-1.88)
Separated/divorced/widowed	115 (21.9)	42 (26.7)	1.37 (0.83,2.24)	85 (20.8)	18 (21.9)	0.88 (0.45-1.71)
Years of education						
≥16 years	146 (27.8)	69 (43.9)	Reference	130 (31.9)	21 (25.6)	Reference
13-15 years	161 (30.7)	48 (30.6)	1.07 (0.66,1.73)	121 (29.7)	32 (39.0)	1.66 (0.90-3.07)
≤12 years	218 (41.5)	40 (25.5)	1.11 (0.69,1.78)	157 (38.5)	29 (35.4)	1.30 (0.67-2.51)
Household income						
≥$90,000	95 (18.1)	19 (12.1)	Reference	89 (21.8)	15 (18.3)	Reference
$50,000-$89,999	127 (24.2)	33 (21.0)	1.36 (0.73,2.55)	109 (26.7)	23 (28.0)	1.38 (0.67-2.81)
$25,000-$49,999	99 (18.9)	33 (21.0)	1.81 (0.95,3.44)	74 (18.1)	19 (23.2)	1.73 (0.81-3.72)
<$25,000	165 (31.4)	65 (41.4)	2.10 (1.16,3.81)	109 (26.7)	24 (29.3)	1.57 (0.75-3.29)
Income for household size[c]						
Above the federal poverty line	395 (75.2)	113 (72.0)	Reference	325 (79.7)	70 (85.4)	Reference
Below the federal poverty line	91 (17.3)	37 (23.6)	1.35 (0.86,2.11)	56 (13.7)	11 (13.4)	0.93 (0.46-1.89)
Health insurance						
Yes	475 (90.5)	133 (84.7)	Reference	379 (92.9)	72 (87.8)	Reference
No	50 (9.5)	24 (15.3)	1.52 (0.89,2.61)	29 (7.1)	10 (12.2)	1.70 (0.75-3.84)
Regular preventive care[d]						
Yes	-	-	-	-	-	-
No	-	-	-	-	-	-

[a] Screening eligibility was defined as 50 or older

[b] ORs were adjusted for age at diagnosis (continuous), income for household size, and insurance status.

[c] Calculation was based off of the 2013 poverty line.

[d] Receipt of regular preventive care prior to breast cancer diagnosis was not queried in the Women's Circle of Health studies.
